# CAFs and TGF-β Signaling Activation by Mast Cells Contribute to Resistance to Gemcitabine/Nabpaclitaxel in Pancreatic Cancer

**DOI:** 10.3390/cancers11030330

**Published:** 2019-03-07

**Authors:** Letizia Porcelli, Rosa Maria Iacobazzi, Roberta Di Fonte, Simona Serratì, Angelica Intini, Antonio Giovanni Solimando, Oronzo Brunetti, Angela Calabrese, Francesco Leonetti, Amalia Azzariti, Nicola Silvestris

**Affiliations:** 1 Experimental Pharmacology Laboratory, IRCCS Istituto Tumori “Giovanni Paolo II”, 70124 Bari, Italy; porcelli.letizia@gmail.com (L.P.); rosamaria.iacobazzi@gmail.com (R.M.I.); difonte.roberta@gmail.com (R.D.F.); simonaserrati@hotmail.com (S.S.); angelicaintini@gmail.com (A.I.); 2 Department of Biomedical Sciences and Human Oncology, Section of Internal Medicine ‘G. Baccelli’, University of Bari Medical School Bari, 70124 Bari, Italy; antoniogiovannisolimando@gmail.com; 3 Medical Oncology Unit, Ospedale Mons. R. Dimiccoli, 76121 Barletta (Bat), Italy; dr.oronzo.brunetti1983@gmail.com; 4 Radiology Unit, IRCCS Istituto Tumori “Giovanni Paolo II”, 70124 Bari, Italy; acalabrese22@gmail.com; 5 Dipartimento di Farmacia-Scienze del Farmaco, University of Bari, 70125 Bari, Italy; francesco.leonetti@uniba.it; 6 Medical Oncology Unit and Scientific Direction, IRCCS Istituto Tumori “Giovanni Paolo II”, 70124 Bari, Italy; n.silvestris@oncologico.bari.it

**Keywords:** CAFs, gemcitabine, mast cells, nabpaclitaxel, pancreatic cancer

## Abstract

Tumor–stroma interactions are of key importance for pancreatic ductal adenocarcinoma (PDAC) progression. Our aim was to investigate whether cancer associated fibroblasts (CAFs) and mast cells (MC) affected the sensitivity of PDAC cells to gemcitabine/nabpaclitaxel (GEM/NAB). For this purpose, the combination cytotoxicity and the effect on tumor invasion and angiogenesis were evaluated with or without a conditioned medium from the mast cell line HMC-1 (human mast cell line-1 cells) and CAFs. Beside the clinical outcome of a homogenous population of PDAC patients, receiving GEM/NAB, was correlated to the circulating levels of mast cell tryptase and to a panel of inflammatory and immunosuppressive cytokines. CAFs neither affected drugs’ cytotoxicity nor the inhibition of angiogenesis, but promoted tumor cell invasion. The MC instead, caused resistance to drugs by reducing apoptosis, by activating the TGF-β signalling and by promoting tumor invasion. Indeed, the inhibition of TβRI serine/threonine kinase activity by galunisertib restored drugs cytotoxicity. Moreover, MC induced the release of TGF-β1, and increased expression of PAR-2, ERK1/2 and Akt activation. Accordingly, TGF-β1, tryptase and other pro-inflammatory and immunosuppressive cytokines increased in the unresponsive patients. In conclusion, MC play a pivotal role in the resistance to GEM/NAB. A correlation between high level of circulating pro-inflammatory/ immunosuppressive cytokines and unresponsiveness was found in PDAC patients.

## 1. Introduction

Pancreatic ductal adenocarcinoma (PDAC) is one of the most aggressive malignancies, with a 5-year overall survival of less than 5%. Depending on patients’ performance status [1,2,3] the most successful chemotherapy regimens are gemcitabine, FOLFIRINOX and gemcitabine/nabpaclitaxel (GEM/NAB). However, the clinical management of patients still remains challenging, because in the majority of cases they present an intrinsic resistance to therapies and currently none of the candidate molecular markers, identified in genome-wide comprehensive studies, have shown an appropriate clinical performance to be routinely used in the management of chemotherapeutics resistance. Pancreatic cancer develops in a microenvironment, the stroma, enriched with extracellular matrix proteins, mainly produced by stellate cells known as cancer-associated fibroblasts (CAFs), inflammatory cells such as mast cells (MC), and small blood vessels that recent evidence suggests are a dynamic compartment rather than a mechanical barrier, strongly involved in the process of tumor formation, progression, invasion and metastasis [4,5]. Indeed the paracrine cross-talk of tumor cells and stroma cells has been demonstrated to play a pivotal role in tumor cells’ transformation and recently even in chemoresistance [6,7]. In vitro evidence suggested that among stroma cells, CAFs played a major role in the acquisition of the hallmarks of pancreatic cancer, including chemoresistance [8,9] whereas the presence of inflammatory cells, such as mast cells infiltrating the pancreatic cancer, has been associated with a worse prognosis, because it promoted angiogenesis, formation of the desmoplastic microenvironment and invasion of the tumor [10,11,12]. Recently, Wroblewski et al. showed that MC reduced the efficacy of anti-angiogenic therapy [13]; however, to the best of our knowledge, to date there is no evidence relating tumor infiltrating mast cells and resistance to chemotherapy, such as GEM/NAB, on pancreatic cancer. Therefore, we hypothesized a potential role of mast cells and CAFs in the resistance to GEM/NAB. To test this hypothesis, we utilized four PDAC cellular models of PDAC treated with this chemotherapy regimen in the presence and absence of the conditioned medium obtained by the HMC-1 (human mast cell line-1 cells) cell line [14] and by CAFs. So far, we have mimicked the crosstalk between tumor cells and stromal cells gaining insights on the mechanisms involved in the resistance to GEM/NAB on cell viability. In addition, we tested the effect of CAFs and MC on GEM/NAB-dependent inhibition of invasion and angiogenesis. Since upon degranulation serine proteases such as tryptase are the major constituent of secretory granules released by mast cells, in addition to cytokines, growth factors and other bioactive molecules [15], we focused on the transforming growth factor-β (TGF-β) signalling and on the G protein-coupled receptor proteinase-activated receptor 2 (PAR2) pathway, that in response to activation by serine proteinases, such as tryptase, drives the progression of pancreatic cancer. This because, when deregulated, the TGF-β signalling [16] plays a crucial role in the paracrine crosstalk between epithelial tumor cells and stroma cells to promote tumor growth, extracellular matrix remodelling, stemness, evasion of immune surveillance and cancer drug resistance [17,18,19,20,21]. Furthermore, increasing evidence suggested a close functional interaction between TGF-β1/ALK5 and PAR2 signalling in regulating tumor-stroma crosstalk in pancreatic cancer [22]. Additionally, because mast cells and CAFs enhance the content of tumor promoting mediators within the tumor microenvironment [23], we assessed the clinical relevance of evaluating the impact of such cells on the response to GEM/NAB combination, by determining in the blood of PDAC patients the levels of tryptase and of 12 pro-inflammatory, immune-suppressive and pro-angiogenic mediators, notoriously altered in PDAC patients and associated with the severity of PDAC [24]. This was done with the aim of suggesting a predictive role for them in the response to GEM/NAB. To this purpose we conducted a prospective study with a well-defined homogenous population, which had a follow-up of two months from the beginning of GEM/NAB treatment, so as to determine a correlation between the levels of tryptase and cytokines with responsive or unresponsive patients.

## 2. Results

### 2.1. Pancreatic Cells’ Sensitivity to Gemcitabine and Nabpaclitaxel

To determine the effectiveness of gemcitabine and nabpaclitaxel on pancreatic cancer cells, we challenged PANC-1 (human PDAC cells) and AsPC-1 (human pancreas adenocarcinoma ascites metastasis cells) with different concentrations of both drugs ranging from 0.1–100 µM, whereas MIA PaCa-2 (cell line from an undifferentiated human pancreatic carcinoma) and CFPAC-1 (human pancreatic cell from liver metastasis) with concentrations ranging from 0.001–10 µM. After a 3-day incubation period, cell proliferation was reduced in a dose-dependent manner allowing determination of 50% inhibition of cell growth (IC_50_) for each drug in each cell line, as reported in Table 1. All experiments were performed in serum-free conditions and repeated at least three times. All results are expressed as mean and standard deviation.

### 2.2. Effectiveness of Gemcitabine/Nabpaclitaxel (GEM/NAB) on Cell Viability and Apoptosis Induction Was Reduced with CM-HMC-1 but Not with CM-CAF

**Figure 1:**
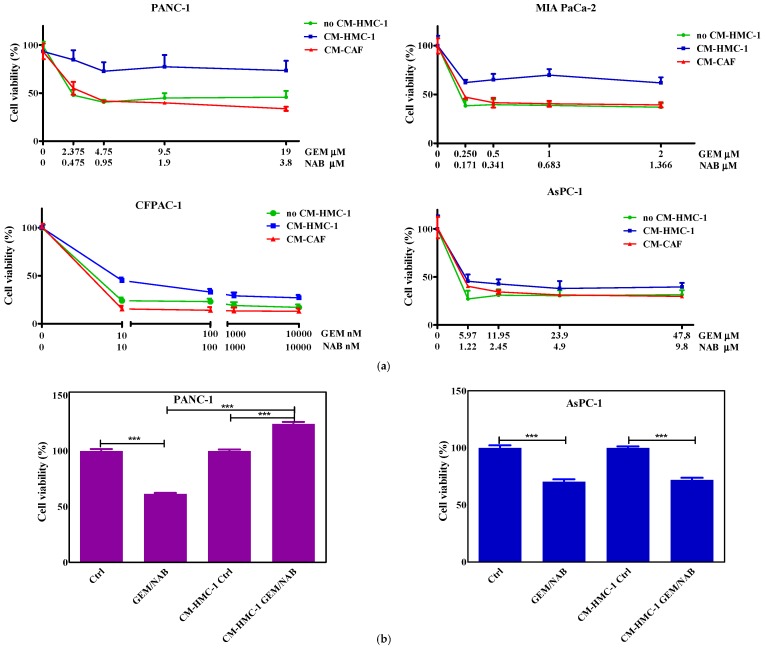
Dose response plots showing the inhibition of cell viability after treatment with GEM/NAB in the presence and absence of CM-HMC-1 or CM-CAF in 2D and 3D culture system. (**a**) PANC-1, MIA PaCa-2 and AsPC-1 cells were incubated with GEM/NAB at concentration: IC_50_/4, IC_50_/2, IC_50_, 2 × IC_50_, in the presence or absence of CM-HMC-1 and CM-CAFs. CFPAC-1 cells were incubated with GEM/NAB at concentration: IC_50_, IC_50_ × 10, IC_50_ × 100, IC_50_ × 1000, in the presence or absence of CM-HMC-1 and CM-CAFs. After 72 h, the survival of cells was determined and results are shown as dose-response plots. The results are the mean of three different experiments. (**b**) 3D cell culture of AsPC-1 and of PANC-1 were incubated with the combination at IC_50_s in the presence or absence of CM-HMC-1 for 72 h. Graph bars report cell viability percentage resulting from the mean of three different experiments (mean ± standard deviation (SD), *** *p* < 0.001).

To test the hypothesis that the crosstalk between mast cells or CAFs and pancreatic cancer affects the response to GEM/NAB, we treated the pancreatic cancer cells with the combination of GEM and NAB in the presence and absence of CM-HMC-1 or CM-CAF and we assessed the cell growth. The combination of IC_50_s drugs inhibited cell proliferation of PANC-1, AsPC-1 and MIA PaCa-2 by about 50% compared to controls, instead by almost 80% of CFPAC-1. The presence of CM-HMC-1 strongly reduced combination effectiveness by about 40% in the whole range of concentrations on PANC-1 and MIA PaCa-2; the effectiveness was reduced by about 20% on CFPAC-1 at IC_50_s and to a lesser extent (10%) at higher concentrations of both drugs (Figure 1a). There was almost no reduction of the drugs’ effectiveness found on AsPC-1 at IC_50_s and higher concentrations in presence of CM-HMC-1 (Figure 1a). The presence of CM-CAF did not affect combination effectiveness in any of the cell lines tested, as reported in the dose-response plots in Figure 1. To further confirm the “protection” by CM-HMC-1 on the drugs’ cytotoxicity, 3D models of PANC-1 (positive model) and AsPC-1 (negative model) were used (Figure 1b). The cytotoxicity of drug combination, as shown in Figure 1b, was strongly reduced only in PANC-1 cells.

**Figure 2:**
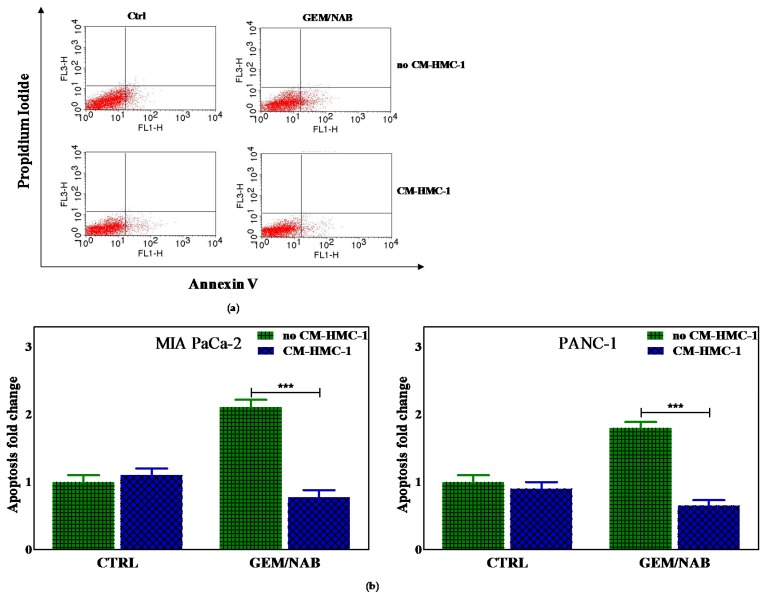
The effect of CM-HCM-1 on drug combination-induced apoptosis by the annexin V method. PANC-1 and MIA PaCa-2 were treated with drug combination with or without CM-HMC-1. After 24 h, the combination induced annexin V staining of tested cells but the apoptosis was completely blocked by the presence of CM-HCM-1. What is shown are (**a**) dot plots from experiments performed on MIA PaCa-2 cells and (**b**) graph bars reporting apoptosis quantification in MIA PaCa-2 and PANC-1 (*** *p* < 0.001).

Subsequently, we explored the effect of CM-HCM-1 on combination-induced apoptosis with the annexin V method. To this purpose all cells were treated with drug combination with or without CM-HMC-1. After 1 day of exposure, the combination induced annexin V staining, which meant the induction of early apoptosis on all cell lines; however the presence of CM-HCM-1 completely blocked GEM/NAB-induced apoptosis only in PANC-1 and MIA PaCa-2 cells. Figure 2a shows a representative analysis of annexin V staining performed in MIA PaCa-2 cells, whereas in Figure 2b the histogram plot reports the data from evaluations on MIA PaCa-2 and PANC-1, demonstrating that the addition of CM-HMC-1 offsets the apoptosis induced by GEM/NAB in such cell lines.

### 2.3. CM-HMC-1 Induced Resistance to GEM/NAB through the Activation of TGF-β Signalling

**Figure 3:**
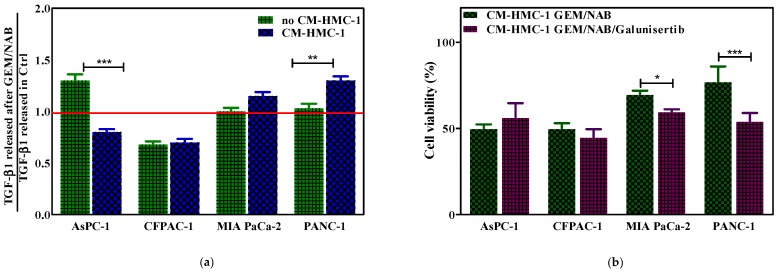
CM-HMC-1 induces the release of TGF-β1 and resistance to GEM/NAB. The release of TGF-β1 from cells was assessed after treatment(s). (**a**) Fold change of TGF-β1 release from GEM/NAB treated cells versus control sample, in presence and absence of CM-HMC-1 (*** *p* < 0.001). (**b**) Selective inhibition of TβRI by galunisertib restored the sensitivity to GEM/NAB in presence of CM-HMC-1. To assess whether TGF-β pathway drives resistance to GEM/NAB, the cell viability was assayed by adding 10 μM galunisertib to CM-HMC-1 + GEM/NAB (*** *p* < 0.001).

Because a significant amount of evidence demonstrated that several chemotherapeutic agents induced autocrine TGF-β1 signalling [21], we assessed the release of TGF-β1 from GEM/NAB-treated cells in the presence and the absence of CM-HMC-1. After three days of treatment TGF-β1 was quantified by a Quantikine enzyme-linked immunosorbent assay (ELISA) in the supernatant of cells. The analysis of the data demonstrated that GEM/NAB induced a 30% increase of TGF-β1 versus the control sample on AsPC-1 (142.16 vs. 109.75 pg/mL), whereas no difference was found on PANC-1 and MIA PaCa-2 treated cells versus control (172.27 vs. 167.63 pg/mL and 154.49 vs. 153.45 pg/mL, respectively). Interestingly, the release of TGF-β1 from GEM/NAB-treated AsPC-1 in the presence of CM-HMC-1 was decreased by almost 20% versus the control sample (109.96 vs. 138.03 pg/mL), indicating that the presence of CM-HMC-1 diminished the release of TGF-β1 from such cells. The opposite effect was observed on PANC-1 and MIA PaCa-2; indeed, when treated with GEM/NAB in the presence of CM-HMC-1, PANC-1 released 30 more TGF-β1 than the control sample (151.65 vs. 116.41 pg/mL and 125.70 vs. 109.30 pg/mL, respectively) and MIA PaCa-2 15% more TGF- β1, suggesting that the presence of CM-HMC-1 induced the autocrine TGF-β1 signalling, which might drive resistance to GEM/NAB in such cells. Unlike PANC-1 and AsPC-1, both the treatment with GEM/NAB and with GEM/NAB + CM-HMC-1, reduced TGF-β1 release of 30% from CFPAC-1 (112.12 vs. 163.96 pg/mL and 131.13 vs. 188.58 pg/mL). These results are summarized in Figure 3a, in which is reported the fold change of TGF-β1 released from GEM/NAB treated cells versus control, in the presence and absence of CM-HMC-1. In order to assess that the autocrine TGF-β signalling activation drives resistance to GEM/NAB, the cells’ viability was determined by adding 10 μM of the TβRI inhibitor galunisertib to GEM/NAB in presence of CM-HMC-1. The addition of galunisertib slightly increased cell viability of AsPC-1, while it restored combination effectiveness on PANC-1 (*** *p* < 0.001) and on MIA PaCa-2 (* *p* < 0.005), and exerted no effect on CFPAC-1 cell viability (Figure 3b).

### 2.4. CM-HMC-1 Induces the Expression of PAR-2 and Activated Erk1/2 and Akt in TGF-β1 Activated Pancreatic Cells

**Figure 4:**
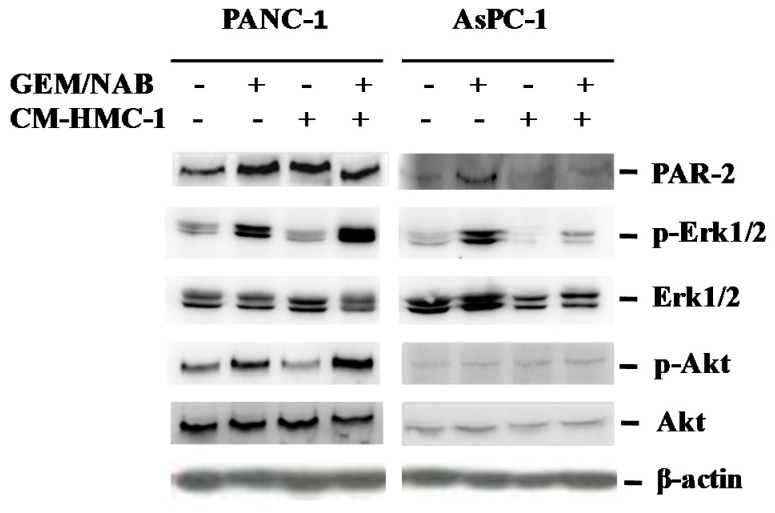
Signalling cascade activated in response to CM-HMC-1 in GEM/NAB treated and untreated cells. PAR-2 expression was increased by GEM/NAB in the responsive cells (AsPC-1) but not when CM-HMC-1 was added; instead in the resistant PANC-1 cells, both GEM/NAB and CM-HMC-1 increased PAR-2 expression. Accordingly, the activation of PAR-2 downstream effector Erk1/2 was found on AsPC-1 after GEM/NAB and on PANC-1 after GEM/NAB and CM-HMC-1, together with the activation of Akt.

In order to understand whether the release of TGF-β1 from PANC-1 treated with GEM/NAB + CM-HMC-1, was due to the activation of TGF-β1 autocrine signalling, we determined the activation status of the SMAD2/3 heteromeric complex. This because characteristically the cellular response to TGF-β1 is the activation of the SMAD pathway through the recruitment, activation and nuclear translocation of the transcription factor SMAD2/3 [25,26]. To this purpose, we performed Western blotting (WB) experiments on cell extract from PANC-1 to verify the activating phosphorylation on SMAD2 (Ser465/467) and on SMAD3 (Ser423/425). However, neither SMAD2 nor SMAD3 were found to be phosphorylated on PANC-1 treated with GEM/NAB + CM-HMC-1.Recently, a functional cooperativity between TGF-β1 and PAR2 has been demonstrated on several cellular responses to tumor microenvironment in PDAC, such as induction of fibrosis, cell motility and invasion [27,28]. Furthermore, TGF-β1 regulates both PAR2 and PAR1 at the transcriptional level with a mechanism dependent on the activation of MAPK such as ERK1/2 and phosphatidyl-inositol-3 kinase (PI3K) but SMAD4-independent [27]. Thus, we investigated whether ERK1/2 and the PI3K effector protein Akt were activated together with the expression level of PAR-2 on TGF-β1 activated cells and not activated ones (AsPC-1 and CFPAC-1) so as to investigate the interplay between PAR-2 and TGF-β signalling pathways. Figure 4 reports the immunoblotting performed on PANC-1 and AsPC-1 treated with GEM/NAB, in the presence and absence of CM-HMC-1 versus untreated cells, showing the activating phosphorylation of Erk1/2, Akt and the expression of PAR-2. We observed on TGF-β1 activated cells (PANC-1), that the treatment with GEM/NAB increased the expression of p-Erk1/2 over untreated cells (3 fold versus 2 fold, respectively) and the combination GEM/NAB + CM-HMC-1 further increased the expression level of p-Erk1/2 up to 5 fold over the control. Accordingly, the expression of PAR-2 in GEM/NAB-treated cells increased more than two fold compared to untreated ones, as well as after CM-HMC-1 and combination condition (GEM/NAB + CM-HMC-1). Moreover both GEM/NAB and the combination condition increased the expression level of p-Akt in these cells. Unlike PANC-1, on AsPC-1 a strong increase of p-Erk1/2 and PAR-2 upon treatment with GEM/NAB, but not when in presence of CM-HMC-1, was found, whereas p-Akt was unaffected either by the drugs and by CM-HMC-1. Unlike PANC-1 and AsPC-1, either the treatment with GEM/NAB and GEM/NAB + CM-HMC-1 induced the activation of the SMAD pathway on CFPAC-1. In fact, we observed an increase of 15% and 40% of p-SMAD3 with GEM/NAB and GEM/NAB + CM-HMC-1 respectively, as compared to each control sample. Accordingly both p-Erk1/2 and p-Akt were increased by GEM/NAB and GEM/NAB + CM-HMC-1 treatments; instead PAR-2 was increased of 20% on GEM/NAB–treated cells versus control sample and not affected by GEM/NAB + CM-HMC-1 versus control sample. This would suggest that the expression of PAR-2 was not correlated to the activation of the TGF-β1 canonical and non-canonical pathway (data reported in Figure S1). 

### 2.5. CM-HMC-1 and CM-CAF Do Not Affect GEM/NAB-Dependent Inhibition of Tumor Angiogenesis but They Reduce GEM/NAB-Dependent Inhibition of Tumor Invasion

**Figure 5:**
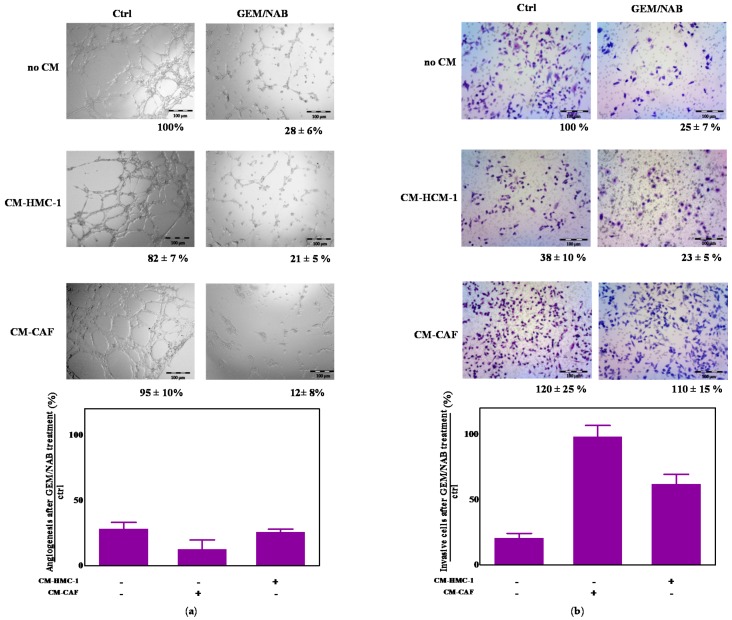
CM-HMC-1 and CM-CAF did not alter GEM/NAB-dependent inhibition of tumor angiogenesis but reduced the efficacy on tumor invasion. (**a**) Capillary morphogenesis. Both CM-HMC-1 and CM-CAF did not alter angiogenesis as demonstrated by microvascular formation and did not reduce the antiangiogenic potential of GEM/NAB. Pictures represent three different experiments. (**b**) Invasion assay. Both CM-HMC-1 and CM-CAF altered tumor invasion, by inducing the decrese and the increase of MIA PaCa-2 invasion, respectively. Both conditioned media reduced the efficacy of GEM/NAB-dependent inhibion of tumor invasion as reported in the histogram plot showing the recovery of tumor invasion on CM-HMC-1/CM-CAF + GEM/NAB treated cells. The pictures represent three different experiments.

Because both mast cells and CAFs favour tumor growth and spread through the release of pro-angiogenic factors and proteases that aid tumor cells’ invasion of the stroma [9], we performed experiments to assess the effect of CM-HMC-1 and CM-CAF on GEM/NAB-dependent inhibition of angiogenesis and tumor invasion. To this end, we performed experiments of capillary morphogenesis on matrigel, by exposing microvascular endothelial cells (H-MVEC, human dermal microvascular endothelial cells) to GEM/NAB in the presence and absence of CM-HMC-1 or CM-CAFs and investigated, through the Boyden chamber invasion assay, the chemotactic effect of both conditioned medium on MIA PaCa-2 treated with GEM/NAB. The pictures representative of three experiments of capillary morphogenesis are reported in Figure 5a, instead of in Figure 5b where the bar graph shows the percentage of residual angiogenesis after treatment with GEM/NAB in presence and absence of CM-HMC-1 or CM-CAF versus control. Both conditioned media exerted no effect on capillary morphogenesis. As reported in the graph, the treatment with GEM/NAB strongly inhibited capillary morphogenesis of H-MVEC (residual angiogenesis of 28 ± 6% versus control) which was not substantially affected by the presence of both conditioned media (21 ± 5% by CM-HMC-1 and 12 ± 5% by CM-CAF). Unlike CM-CAFs, which induced a non-significant increase of tumor cell invasion, the presence of CM-HMC-1 reduced Mia PaCa-2 invasion, through Matrigel-coated filters, of 50% vs. control sample, consisting of a medium that was not conditioned. However, CM-HMC-1 strongly reduced the GEM/NAB-dependent inhibition of tumor cells’ invasion and CM-CAFs completely abrogated it.

### 2.6. Tryptase and Cytokines’ Modulation in Pancreatic Ductal Adenocarcinoma (PDAC) Patients

The level of some soluble mediators, released from tumor and stromal cells, were measured in plasma or serum samples obtained from 10 advanced PDAC patients, whose clinical pathological features are reported in Table 2.

**Figure 6:**
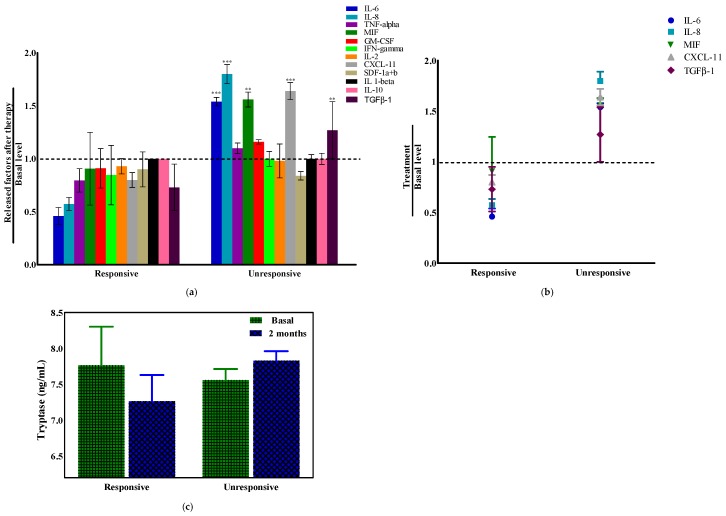
Tryptase and cytokines released in the blood of patients according to response to therapy. The analysis of some mediators released from tumor and stromal cells were measured in plasma or serum samples of 10 advanced pancreatic ductal adenocarcinoma (PDAC) patients. Venous blood was drawn before therapy (baseline) and after 2 months. (**a**) Graph bars report the fold change of cytokines in blood samples in responsive and unresponsive PDAC patients (**b**) the chart reports significantly different cytokine levels among the groups of patients. Statistical significance has been calculated using a two-way analysis of variance (ANOVA) followed by the Bonferroni post hoc tests (GraphPad Prism vers. 5). Data were indicated with ** *p* < 0.01, and *** *p* < 0.001. (**c**) Graph bars reports plasma levels of tryptase in PDAC patients.

It is known that the increase in the level of cytokines together with other factors such as reactive oxygen and nitrogen species in tumor microenvironment, promote inflammation-mediated events contributing to pancreatic cancer development, progression and invasion [29]. To further investigate the role that tumor–stroma interactions can have on the response to GEM/NAB treatment in PDAC, we evaluated the levels of several cytokines involved in inflammation, immunosuppression and angiogenesis in pancreatic cancer [24] (GM-CSF, IFN-gamma, IL-10, IL-1beta, IL-2, IL-6, IL-8/CXCL8, I-TAC/CXCL11, MIF, SDF-1a + b/CXCL12, TNF-alpha, and TGF-β1) and of mast cell tryptase in the blood of PDAC patients. Among them, only the Macrophage Migration Inhibitory Factor MIF and CXCL11 significantly varied between the group of responsive and unresponsive patients at basal level, meaning before the start of the therapy. Indeed MIF, which is also correlated with resistance to GEM [30], was of about 1300 pg/mL in the group of responsive patients, whereas it was markedly increased (about 5500 pg/mL) in the group of unresponsive patients. CXCL-11 chemokine, notoriusly involved in immune response [31], was about 23 pg/mL at basal level in the responsive group and reduced to 12.6 pg/mL in the unresponsive one. From the analysis of the cytokines, we identified five of them (IL-6, IL-8, MIF, TGF-β1 and CXCL-11) which were significantly altered (increased) in PDAC patients’ serum and correlated with unresponsiveness to GEM/NAB. Figure 6b shows the fold change of all cytokines, obtained from the ratio between the level after two months therapy and the basal level, in the responsive group versus the unresponsive group. In Figure 6b the scatter plot summarizes the fold change of the five cytokines which had significantly increased in the unresponsive group versus the responsive one. Our results further strengthen recent findings [32,33], suggesting that monitoring the plasma/serum level of cytokines might be helpful in the identification of diagnostic, predictive and prognostic biomarkers for PDAC. Regarding mast cell tryptase, from the evaluation of blood level before and after two months of therapy, we found that it was reduced in the responsive group while it increased in the unresponsive one (Figure 6c).

## 3. Discussion

The main result of our study is that for the first time we demonstrated that the crosstalk between mast cells and PDAC cells strongly reduced the GEM/NAB-dependent inhibition of tumor cell viability through the activation of TGF-β signalling. Indeed the selective inhibition of the type I TGF-β1 receptor (ALK5) serine/threonine kinase by galunisertib restored the sensitivity to GEM/NAB in the presence of CM-HMC-1, because it interrupted the crosstalk with such stoma cells. It is noteworthy that a significant protection from GEM/NAB cytotoxicity was observed only with the presence of CM-HMC-1 and not with CM-CAFs, suggesting that the combination GEM/NAB is endowed with anti-stromal activity which completely counteracted pro-survival signals engaged by CAFs. This finding is in agreement with the stromal-disrupting effects of nabpaclitaxel documented by Alvarez et al. [34], who demonstrated that thanks to nabpaclitaxel the patients treated with the combination GEM/NAB displayed a softer stroma than patients treated with gemcitabine alone, because it became less abundant in CAFs and the collagen fibers were disrupted and disorganized, thus determining tumor softening. This evidence prompted us to investigate signal transduction pathways crucial in the crosstalk between tumor cells and stroma, such as TGF-β and PAR-2 signalling, notoriously involved in pro-fibrotic/pro-inflammatory process in pancreatic cancer [35], as inducers of resistance to GEM/NAB.

The release of TGF-β1 from tumor cells has been suggested as a mechanism of resistance to chemotherapy [21]. We found that the levels of TGF-β1 released from cells after GEM/NAB, decreased with the addition of CM-HMC-1 in cells which did not develop resistance to therapy (AsPC-1/CFPAC-1); the opposite effect was observed on cells which instead became resistant (PANC-1/MIA PaCa-2). It is interesting to note that even PAR-2 was differentially expressed between cells after treatments. Considering the release of TGF-β1, it has been reported that by stimulating HaCaT immortalized keratinocyte cell line with trypsin, a serine protease-like tryptase released by mast cells, TGF-β1 was released from cells through a mechanism dependent on PAR-2-mediated EGFR transactivation and ERK1/2 signalling activation [36]. The activation of ERK1/2 has been demonstrated to play a crucial role in the TGF-β dependent malignant transformation of pancreatic cancer [37]; this is because KRAS mutations and altered TGF-β signalling are frequent in pancreatic cancer [38]. Thus, perhaps on cells which developed resistance to GEM/NAB in presence of CM-HMC-1, a complex autocrine signalling loop occurred which started with the activation of PAR-2 by tryptase and involved the TGF-β receptor and maybe other tyrosine kinase receptors, through transactivation, and this has led to TGF-β1 release, PAR-2 increased expression and ERK signalling activation, which ultimately drove tumor cells’ proliferation and resistance to treatment. The functional crosstalk between PAR-2 and TGF-β1 signalling in resistant cells is further suggested by the activation of Akt, which is another downstream mediator of PAR-2 and of the deregulated TGF-β pathway in pancreatic cancer [39]. In addition, recent findings showed that TGF-β1 regulated the expression of PAR-1/2, whereas PAR-2 triggered TGF-β1 release [22].

A great body of evidence has demonstrated that chemotherapeutic drugs remodel the tumor microenvironment by promoting the release of pro-inflammatory and immunosuppressing cytokines, which promote tumor re-growth and drug resistance [40].

In our study, by mimicking the crosstalk with mast cells we found that GEM/NAB promotes the release of TGF-β1 from tumor cells, and we suggested that in cells in which a functional interplay between TGF-β1 and PAR-2 signalling occurs, resistance to GEM/NAB develops.

Furthermore, by clustering the patients enrolled in a prospective study in responsive and unresponsive group, we found that the unresponsiveness to GEM/NAB correlated with a significant increase of TGF-β1, IL-6, IL-8, MIF, and CXCL-11 in the blood of patients and with an increase of tryptase. Notoriously the release of TGF-β1, IL-6 and IL-8 are under the control of PAR-2 and TGF-β1 signalling, whereas increased MIF and CXCL-11 account for the development of an immunosuppressive microenvironment, which is characteristic of a tumor with a strong activation of ERK1/2 [41] and TGF-β1 signalling deregulation. Despite the fact that the level of tryptase did not reach a significant p value, the aforementioned results strongly suggest that the clinical impact of mast cells goes beyond their recognized role in tumor prognosis, because they might play a crucial role in the resistance to GEM/NAB.

To broaden the evaluation of the crosstalk between mast cells or CAFs and pancreatic cancer, we also tested their effect on GEM/NAB-dependent inhibition of tumor invasion and angiogenesis. We found that while CM-CAFs promoted MIA PaCa-2 invasion, on the other hand mast cells prevented it. However both conditioned mediums reduced the anti-invasive activity of GEM/NAB. No significant effect was observed on GEM/NAB-dependent inhibition of angiogenesis by both cell types. Our results, which show that mast cells reduced MIA PaCa-2 invasion, do not seem to agree with the results reported by Strouch et al. [10], who conversely reported that human mast cells LAD-2 promoted the invasion of PANC-1 and AsPC-1. However we are convinced that the experimental conditions in which the experiments were carried out (48 h against 24 h in our experiments) could account for the different effects observed. Indeed, by increasing the time to perform Boyden chamber matrigel invasion assay, the activation of tumor invasion by mast cells should not be excluded.

## 4. Materials and Methods

### 4.1. Cell Culture

PANC-1 human PDAC cells, AsPC-1 human pancreas adenocarcinoma ascites metastasis cells, MIA PaCa-2 cell line from an undifferentiated human pancreatic carcinoma, CFPAC-1 human pancreatic cell from liver metastasis, were purchased from ATCC ^®^-LGC standards, (Teddington Middlesex, UK). HMC-1 human mast cell line-1 cells were kindly provided by Prof. L. Macchia, University of Bari, CAF cells were purchased from Vitro Biopharma, and MVEC human dermal microvascular endothelial cells were purchased from Lonza, Switzerland. All cell lines were grown as recommended by the supplier. The conditioned medium (CM) of HMC-1 and of CAF cells was collected every 24 h and diluted to 1/3 with the typical growth medium of each cell line before using it. All materials for cell culturing were purchased from EuroClone, Italy.

### 4.2. 3D Cell Culture

PANC-1 and AsPC-1 cells were seeded on 12-well inserts Alvetex^®^ Scaffold (REPROCELL Europe Ltd., Glasgow, UK) at a density of 300,000 cells/well and grown in complete Dulbecco’s modified Eagle’s medium (DMEM) for 10 days in order to achieve a 3D scaffold. Afterwards, cells were treated with the combination GEM/NAB (IC_50_) in CM-HMC-1 cells for further 72 h and then recovered following the protocol described by the manufacturer (Consolidated Protocol Booklet. Reinnervate, The Real 3D Cell Culture, REPROCELL Europe Ltd., Glasgow, UK).

### 4.3. Cell Proliferation Assay

The 3-[4,5-dimethylthiazol-2-yl]-2,5-diphenyltetrazoliumbromide (MTT) assay was performed to investigate the effect of GEM, NAB and their combination in presence and in absence of conditioned medium of HMC-1 cells or CAFs on cell viability of PANC-1, AsPC-1, MIA PaCa-2 and CFPAC-1 cell lines. The results are shown as cell viability (%)/dose plots or histograms of the mean of three different experiments.

#### 4.3.1. IC_50_ Determination

Cells were seeded in 96 wells plates at a density of 5000 cells/well and after their attachment treated for 72 h with drugs in four different concentrations for each cell line. The concentrations of each drug responsible for 50% inhibition of cell growth (IC_50_) were calculated from dose-response curves using non linear multipurpose curve fitting program CalcuSyn [42].

#### 4.3.2. Combination Study

Cells were seeded in 96 wells plates at a density of 5000 cells/well and after their attachment treated for 72 h with GEM and/or NAB, utilizing four drug concentrations (IC_50_/4, IC_50_/2, IC_50_, 2 × IC_50_) in the presence or absence of CM-HMC-1 or CM-CAF on PANC-1, AsPC-1 and MIA PaCa-2 and on CFPAC-1 (IC_50_, IC_50_ × 10, IC_50_ × 100, IC_50_ × 1000). Furthermore, the effect of 10 µM galunisertib on the proliferation of cells treated with the combination GEM/NAB, plus the CM-HMC-1, was evaluated with MTT assay after 72 h of incubation.

#### 4.3.3. 3D Characterization

Cells were cultured as previously described; after 3 days exposure to drugs in the absence or in the presence of CM-HMC-1, MTT cell viability assay was performed as recommended by the manufacturer.

### 4.4. Cell Cycle Analysis

For the cell cycle analysis, human pancreatic cancer cells were seeded in 60 mm dishes at a density of 500,000/well and incubated for 1 day at 37 °C with drug(s) at the IC_50_ concentration. Afterward cells were harvested, washed twice in ice-cold phosphate buffered saline (PBS) pH 7.4, fixed in 4.5 ml of 70% ethanol and stored at −20 °C until analysis. Fixed cells were processed as previously described [43]. Cell cycle determinations were performed using a FACScan flow cytometer (Becton Dickinson, Franklin Lakes, NJ, USA), and data were interpreted using the Cell Quest software, provided by the manufacturer.

### 4.5. Cell Apoptosis Assay

The FITC Annexin V Apoptosis Detection Kit II (BD Pharmingen^TM^–BD Biosciences, Erembodegem, Belgium,) was used in order to detect the number of apoptotic cells after treatment of human pancreatic cancer cell with the combination GEM/NAB (IC_50_ values) in presence of the CM-HMC-1 [44]. The cells were processed according to the instructions provided by the manufacturer.

### 4.6. Detection of Mast Cells-Released Tryptase and TGF-β1 by Enzyme-Linked Immunosorbent Assay (ELISA) Kit

The levels of TGF-β1 was evaluated in PDAC patients’ plasma specimens and in PDAC cells, (after treatment for 72 h with GEM/NAB and CM-HMC-1) through the Human TGF-β1 Quantikine^®^ ELISA (R&D SISTEMS^®^, Abingdon, UK), whereas the level of tryptase on PDAC patients’ serum was evaluated with the Human Mast cell Tryptase (MCT) ELISA kit (MyBioSource, San Diego, CA, USA) and respectively, according to the manufacturer’s instructions.

### 4.7. Bio-PlexProTM Human Chemokine Assay

The level of different cytokines (GM-CSF, IFN-gamma, IL-10, IL-1beta, IL (GM-CSF, IFN-gamma, IL-10, IL-1beta, IL-2, IL-6, IL-8/CXCL8, I-TAC/CXCL11, MIF, SDF-1a + b/CXCL12 and TNF-alpha) released in blood of PDAC patients at basal level and after two months of therapy, was evaluated according to the manufacturer’s instructions.

### 4.8. Western Blotting (WB) Analysis

After treatment of human pancreatic cancer cells for 24–72 h with NAB, GEM and their combination, both in presence and in absence of CM-HMC-1, the protein extracts were obtained by homogenization in RIPA buffer and treated with 1 mM phenylmethylsulfonyl fluoride (PMSF). The protein level of the selected proteins (pErk1/2 Erk1/2, pSMAD3, pAkt (Ser473), Akt, was analyzed by WB as previously described [44]. The blot detection was performed with ChemiDoc™ Imaging Systems and analyzed with the ImageLab software (version, Bio-Rad-USA, Hercules, CA, USA). Antibodies: the monoclonal antibody anti Erk1/2, p-Erk1/2, anti Akt, anti p-Akt(Ser473) were provided by Cell Signalling-USA and anti-β-actin (AC-15) by Sigma-Aldrich. A mouse-HRP and a rabbit-HRP (Bio-Rad Laboratories, Hercules, CA, USA) were used as secondary antibody. All experiments were performed in triplicate.

### 4.9. Invasion Assay

MIA PaCa-2 cells invasion was studied in Boyden chambers as previously described [45]. Briefly, MIA PaCa-2 cell (3 × 10^4^) were placed in the upper well of the chamber, and invasion was performed for 24 h at 37 °C in 5% CO_2_. To evaluate the effects of conditioned media of HMC-1 and CAFs respectively, we measured MIA PaCa-2 cell invasion both as direct conditioned medium–stimulated invasion (chemoinvasion) [conditioned medium in lower well] and in combination with GEM/NAB.

### 4.10. In Vitro Capillary Morphogenesis Assay

The ability of MVEC to move within ECM in presence of the conditioned medium of HMC-1 and CAFs, with or without GEM/NAB, was evaluated as described in [46]. Matrigel (50 µL; 10–12 mg/mL) was pipetted into 0.64-cm (diameter) tissue culture wells and polymerized for 30 min to 1 h at 37 °C. MVEC were plated (12 × 10^3^/mL), in EGM-2 (Endothelial Growth Medium, EGM™-2MV BulletKit™ Lonza, Switzerland) 2% fetal bovine serum (FBS). The effects on morphogenesis of endothelial cells were recorded after 6 h with an inverted microscope. Results were quantified at 6 h by measuring the percentage of field occupancy of capillary projections. Six to nine photographic fields from three plates were scanned for each point.

### 4.11. Study Population

This is a prospective study enrolling patients from the IRCCSIstituto Tumori “Giovanni Paolo II”, Bari, Italy. We enrolled 10 consecutive patients with histologically diagnosed PDAC and treated with a GEM/NAB combinations as first-line chemotherapy.

The study was approved by the ethics committee of the NCRC Istituto Tumori “Giovanni Paolo II” (n. 566/CE), and written informed consent was obtained from all the patients enrolled in the study. All the records were reviewed and the following data were collected: gender (male vs. female), age (< vs. ≥ 65 years), Eastern Cooperative Oncology Group Performance Status (ECOG PS) (0, 1 and 2) and response to chemotherapy according to the RECIST 1.1 criteria (complete response, CR; partial response, PR; stable disease, SD; progressive disease, PD). Venous blood was drawn before therapy (baseline) and after 2 months (for all the patients). Blood samples were centrifuged after 0/30 min at 4 °C and plasma and serum fractions were divided in aliquots, frozen and stored at −80 °C until assayed.

### 4.12. Statistical Analysis

Statistical significance has been calculated using a two-way analysis of variance (ANOVA) followed by the Bonferroni post hoc tests (GraphPad Prism vers. 5). Data were indicated with ** *p* < 0.01, and *** *p* < 0.001.

## 5. Conclusions

Resistance to chemotherapy heavily affects the clinical outcome of patients. Herein, we first uncovered a novel role for mast cells in the resistance to GEM/NAB by demonstrating that, through the activation of TβRI signalling, they promoted tumor cells’ re-growth. These findings further support the clinical use of galunisertib in combination with chemotherapy as already demonstrated by Melisi et al., who showed that galunisertib–gemcitabine vs. gemcitabine improved overall survival in patients with unresectable pancreatic cancer [47]. Additionally we showed that both CAFs and MC reduced the effectiveness of GEM/NAB on tumor invasion. Moreover in accordance with emerging evidence indicating that the identification of circulating biomarkers maybe a better tool for discriminating patients who are likely to benefit from treatment with chemotherapy, we provided a proof of concept that the dosage of level of MIF, IL-6, IL-8, CXCL11, TGF-β1 and tryptase after early time periods from the beginning of therapy could allow monitoring of the response to GEM/NAB and eventually indicate treatment modifications.

## Figures and Tables

**Table 1:** IC_50_ values of pancreatic cancer cells treated with gemcitabine and nabpaclitaxel.

IC_50_ (µM) ^1^		
Cell Line	GEM	NAB
AsPC-1	23.9 ± 2.1	4.9 ± 1.1
PANC-1	9.5 ± 0.8	1.9 ± 0.4
MIA PaCa-2	1 ± 0.15	0.683 ± 0.104
CFPAC-1	0.007 ± 0.003	0.008 ± 0.005

^1^ Cells were seeded in 96 wells plates at a density of 5000 cells/well and after their attachment they were treated for 72 h with gemcitabine (GEM) and nabpaclitaxel (NAB) in six different concentrations for each cell line. The concentrations of each drug yielding 50% inhibition of cell growth (IC_50_) were calculated from dose-response curves using Calcusyn software.

**Table 2:** Patients Characteristics (*n* = 10).

Patients Characteristics	Frequency (%) or Median (IQ)
Age	64.7
Gender	
Male	7 (70%)
Female	3 (30%)
Primary tumor location	
Head	7 (70%)
Body/tail	3 (30%)
Stage at diagnosis	
IV stage (TMN 7th edition)	10 (100%)
Site of metastasis	
Liver	6 (60%)
Lung	1 (10%)
Nodes	7 (70%)
Peritoneum	2 (20%)
Other	1 (10%)
ECOG PS	
0	3 (30%)
1	6 (60%)
2	1 (10%)
First line therapy	
GEM/NAB	10 (100%)
First line PFS	
<6 months	4 (40%)
>6 months	6 (60%)
Response to first-line CT	
Responders (CR, PR, SD)	6 (60%)
Non Responders (Pro)	4 (40%)

(*n*, number; IQ, inter-quartile; TNM, tumor-node-metastasis; ECOG PS, eastern cooperative oncology group performance status; PFS, performance free survival; CT, chemotherapy; CR, complete response; PR, partial response; SD, stable disease; Pro, progression).

## Supplementary Materials

The following are available online at https://www.mdpi.com/2072-6694/11/3/330/s1, Figure S1: Signalling cascade activated in response to CM-HMC-1 in GEM/NAB treated and untreated CFPAC-1 cells. The treatment with GEM/NAB and GEM/NAB + CM-HMC-1 induced the activation of SMAD pathway on CFPAC-1 with an increase of 15% and 40% of p-Smad3 with GEM/NAB and GEM/NAB + CM-HMC-1, respectively, versus each control. p-Erk1/2 and p-Akt were increased by GEM/NAB and GEM/NAB + CM-HMC-1 treatments; PAR-2 was increased of 20% on GEM/NAB treated cells versus control and not affected by GEM/NAB + CM-HMC-1 versus control.
