# Supplementary material for: CAFs and TGF-β Signaling Activation by Mast Cells Contribute to Resistance to Gemcitabine/Nabpaclitaxel in Pancreatic Cancer
Source: Cancers (Basel). 2019 Mar 7;11(3):330. doi: 10.3390/cancers11030330 (PMC6468868; doi:10.3390/cancers11030330)
Supplement: Supplementary file 1 [file cancers-11-00330-s001.pdf]

Supplementary Materials: CAFs and TGF-β Signaling Activation by Mast Cells Contribute to Resistance to Gemcitabine/Nabpaclitaxel in Pancreatic Cancer

Letizia Porcelli, Rosa Maria Iacobazzi, Roberta Di Fonte, Simona Serratì, Angelica Intini, Antonio Giovanni Solimando, Oronzo Brunetti, Angela Calabrese, Francesco Leonetti, Amalia Azzariti and Nicola Silvestris

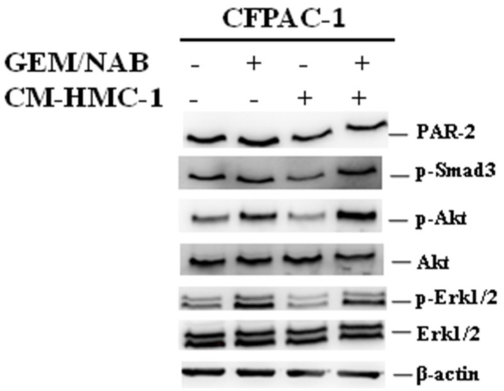

Figure S1: Signalling cascade activated in response to CM-HMC-1 in GEM/NAB treated and untreated CFPAC-1 cells. The treatment with GEM/NAB and GEM/NAB + CM-HMC-1 induced the activation of SMAD pathway on CFPAC-1 with an increase of 15% and 40% of p-Smad3 with GEM/NAB and GEM/NAB + CM-HMC-1, respectively, versus each control. p-Erk1/2 and p-Akt were increased by GEM/NAB and GEM/NAB + CM-HMC-1 treatments; PAR-2 was increased of 20% on GEM/NAB treated cells versus control and not affected by GEM/NAB + CM-HMC-1 versus control.

© 2019 by the authors. Licensee MDPI, Basel, Switzerland. This article is an open access article distributed under the terms and conditions of the Creative Commons Attribution (CC BY) license (http://creativecommons.org/licenses/by/4.0/).
